# The gut microbiome in colorectal cancer: molecular paradigms and translational frontiers

**DOI:** 10.3389/fbioe.2026.1864299

**Published:** 2026-06-16

**Authors:** Yang Liu, Di Mei

**Affiliations:** 1 Department of Laboratory Medicine, Huludao Central Hospital, Huludao, China; 2 Department of Lianshan Gastrointestinal, Hepatobiliary & Bariatric-Metabolic Surgery, Huludao Central Hospital, Huludao, China

**Keywords:** bacteriophages, colorectal cancer, fecal microbiota transplantation, *Fusobacterium nucleatum*, gut microbiome, organoids, tumor microenvironment

## Abstract

The gut microbiome is now recognized as a causal driver of colorectal cancer (CRC) rather than a mere commensal ecosystem. This review elucidates the molecular mechanisms of keystone pathogens, specifically *Fusobacterium nucleatum*, *pks* ^*+*^ *Escherichia coli*, and enterotoxigenic *Bacteroides fragilis*, which induce DNA interstrand crosslinks, hyperactivate Wnt/β-catenin signaling, compromise the epithelial barrier, and reshape the tumor immune microenvironment. We discuss how bioengineered human organoids and microfluidic Organ-on-a-Chip platforms resolve the aerobic-anaerobic co-culture paradox, enabling patient-specific mechanistic dissection of host-microbe crosstalk. From a clinical perspective, we evaluate multi-omics signatures for noninvasive screening, intratumoral bacterial load as a prognostic indicator, and emerging therapeutic strategies including narrow-spectrum antimicrobials, bacteriophage-guided drug delivery, fecal microbiota transplantation for immunotherapy sensitization, and engineered living probiotics. By integrating mechanistic paradigms, organoid-guided validation, and translational applications, we delineate actionable trajectories for precision microbiome targeting in CRC management.

## 1 Introduction

Colorectal cancer (CRC) continues to present a formidable and escalating challenge to global public health. According to the latest GLOBOCAN epidemiological data, CRC ranks as the third most frequently diagnosed malignancy and the second leading cause of cancer-related mortality worldwide, accounting for over 1.9 million new cases and 930,000 deaths annually (Bray et al., 2024). While widespread implementation of colonoscopy screening protocols has yielded a modest decline in incidence among older demographics in highly developed nations, a highly concerning epidemiological paradox has emerged: the incidence of early-onset colorectal cancer (EOCRC), defined as CRC diagnosed in individuals under 50, is rising at an alarming rate globally (Siegel et al., 2023). The etiology of EOCRC is distinct; only a small fraction (approximately 10%–20%) can be attributed to hereditary syndromic mutations such as Lynch syndrome or familial adenomatous polyposis (FAP). The disproportionate surge of sporadic EOCRC strongly implicates the profound influence of modern environmental exposures, specifically Westernized diet, widespread antibiotic utilization, and sedentary lifestyles, acting as primary catalysts for oncogenesis (Akimoto et al., 2021).

For decades, the conceptual framework of CRC pathogenesis has been elegantly codified by the classical “adenoma-carcinoma sequence” delineated by Fearon and Vogelstein. This host-centric genomic model posits that colorectal tumors arise from normal epithelium through the stepwise, temporal accumulation of somatic genetic mutations in critical tumor suppressor genes (e.g., *APC*, *TP53*, *SMAD4*) and oncogenes (e.g., *KRAS*, *BRAF*), coupled with chromosomal instability or microsatellite instability (Fearon and Vogelstein, 1990). While this foundational genomic paradigm remains indisputably relevant, it intrinsically fails to fully elucidate the profound spatial heterogeneity of CRC, nor does it address the complex, dynamic triggers that initiate the earliest *APC* mutations in functionally normal crypts. Consequently, contemporary oncological research has undergone a radical paradigm shift, expanding its focus from a purely host-intrinsic genomic perspective to an integrated, microenvironmental model. At the epicenter of this tumor microenvironment resides the human gut microbiome.

The human gastrointestinal tract harbors a vast, immensely complex ecological community comprising trillions of commensal bacteria, archaea, fungi, and viruses, collectively encoding a genome over 100 times larger than the human genome itself. Under physiological homeostasis, the colonic microbiota maintains a mutualistic symbiosis with the host. It fortifies the intestinal epithelial barrier, synthesizes essential micronutrients and immunomodulatory metabolites, most notably short-chain fatty acids (SCFAs) such as butyrate, which serve as the primary energy source for colonocytes, and continuously educates the mucosal immune system to prevent opportunistic infections (Mann et al., 2024; Kanimozhi et al., 2026). However, the tipping point from mucosal health to malignancy is frequently initiated by “dysbiosis”, a persistent, detrimental perturbation in microbial composition, diversity, and metabolic output.

To conceptualize the temporal dynamics of dysbiosis in oncogenesis, the seminal “driver-passenger” model was introduced (Tjalsma et al., 2012; Chakraborty et al., 2025). This model elegantly postulates that specific indigenous bacteria act as initial “drivers” of carcinogenesis. Pathogens such as genotoxic pks^+^ *Escherichia coli* and Enterotoxigenic *Bacteroides fragilis* (ETBF) invade the protective mucus layer, inducing acute epithelial DNA damage and provoking a cascade of mucosal inflammation. This resulting inflammatory and highly oxidative microenvironment becomes intensely toxic to beneficial commensals but provides a selective survival advantage to opportunistic “passenger” bacteria. These passengers, most notably *Fusobacterium nucleatum* and *Peptostreptococcus anaerobius*, subsequently colonize the nascent adenoma, rapidly outcompeting the original drivers and aggressively fueling malignant progression, immune evasion, and ultimately, metastasis (Wong and Yu, 2023).

Recent advancements in deep shotgun metagenomic sequencing have pushed this paradigm from broad, phylum-level associations to high-resolution, strain-specific causality. For instance, Zepeda-Rivera et al. has demonstrated that *F. nucleatum* is not functionally monolithic; rather, a specific sub-lineage, *F. nucleatum* Clade 2, possesses unique genetic and metabolic adaptations that uniquely equip it to endure gastrointestinal transit and selectively dominate the human colorectal tumor niche (Zepeda-Rivera et al., 2024). Such discoveries demonstrate that the microbiome is not merely an innocent bystander or a secondary consequence of the tumor niche, but an active, indispensable participant in the neoplastic cascade.

Despite robust multi-cohort metagenomic and metabolomic data validating these microbial signatures across diverse global populations (Thomas et al., 2019), bridging the epistemological gap between statistical correlation and definitive mechanistic causality has been historically stymied by profound methodological bottlenecks. Traditional *in vitro* models, such as two-dimensional (2D) immortalized colon cancer cell lines (e.g., Caco-2, HCT116), are severely reductionist. They inherently lack the complex 3D spatial architecture, the crypt-villus axis, cellular heterogeneity, and essential physiological gradients, such as the protective mucin layer and oxygen tension gradients, found in the native human gut (Ternes et al., 2020). When bacteria are co-cultured with 2D monolayers, they often indiscriminately overgrow and induce rapid host cell apoptosis, precluding the study of long-term chronic interactions that characterize true oncogenesis.

Conversely, *in vivo* murine models, while offering the advantage of a systemically intact immune system, possess critical biological divergences. The murine gastrointestinal tract differs fundamentally from the human colon in terms of anatomical structure, baseline mucosal immunology, and, most crucially, the indigenous microbial composition (Nguyen et al., 2015). Many keystone human pathogens fail to stably colonize the murine gut without drastic antibiotic pre-conditioning or germ-free derivation, which artificially skews host-pathogen dynamics. The inability of these conventional platforms to faithfully recapitulate the intricate, human-specific dialogue between the colonic epithelium and the microbiota has necessitated a technological revolution.

The advent of three-dimensional (3D) intestinal organoids has elegantly surmounted these historical constraints. Pioneered by the Clevers laboratory, human intestinal organoids are self-organizing, self-renewing micro-tissues derived from Lgr5^+^ adult stem cells (ASCs) or induced pluripotent stem cells (iPSCs) (Sato et al., 2011). Cultured within a specialized extracellular matrix and defined niche factors, these organoids faithfully preserve the precise genetic and epigenetic signatures of their human donors while differentiating into the full physiological repertoire of intestinal epithelial lineages. Through innovative bioengineering techniques, ranging from luminal microinjection to the generation of apical-out organoids, researchers can now precisely dissect host-microbiome interactions in a highly controlled, patient-specific manner (Pleguezuelos-Manzano et al., 2020). Recent studies have further demonstrated that *E. coli* CNF1 toxin induces fetal reprogramming of intestinal stem cells via Yap/Taz-dependent pathways, providing unprecedented mechanistic insights into how bacterial effectors drive serrated CRC pathogenesis in organoid models (Letourneur et al., 2025).

In light of these rapid conceptual and technological breakthroughs, this review aims to critically evaluate the microbiome-CRC axis. We will first decipher the mechanistic paradigms of microbial oncogenesis, incorporating recent discoveries in subspecies-level pathogenicity and stem cell reprogramming. Subsequently, we will systematically explore the bioengineering evolution of organoid platforms, from basic 3D structures to complex, microfluidic “Organ-on-a-Chip” systems. Finally, we assess the translational landscape, surveying multi-omics diagnostic signatures and critically evaluating emerging microbiome-targeted therapeutics, from precision antimicrobials to engineered probiotics.

## 2 Oncogenic mechanisms of gut microbiota in CRC

To establish unequivocal causality in microbe-driven oncogenesis, it is imperative to move beyond broad taxonomic associations and rigorously decode the specific molecular arsenals deployed by keystone pathogens. The transition from a homeostatic intestinal epithelium to a malignant phenotype is not a passive event but rather an active, pathogen-orchestrated subversion of host cellular machinery. These sophisticated mechanisms can be systematically categorized into direct genotoxicity, aberrant host signaling activation via adhesion, toxin-mediated epithelial barrier subversion, profound remodeling of the tumor immune microenvironment, and the oncogenic modulation of microbial metabolites. Table 1 summarizes the key CRC-associated pathogens and their molecular mechanisms.

**TABLE 1:** Keystone pathogens in colorectal cancer: Molecular mechanisms and clinical implications.

Pathogen	Virulence factor	Molecular target	Major oncogenic mechanism	Clinical association	References
pks^+^ *E. coli*	Colibactin	DNA adenine residues	DNA interstrand crosslinks → DSBs → SBS88 mutational signature	Present in ∼20% of Western CRCs	Pleguezuelos-Manzano et al. (2020), Arthur et al. (2012), Bossuet-Greif et al. (2018), Nougayrède et al. (2006)
*F. nucleatum* Clade 2	FadA, Fap2	E-cadherin, TIGIT	Wnt/β-catenin activation; NK cell inhibition	Advanced stage, poor prognosis, chemoresistance	Zepeda-Rivera et al. (2024), Abed et al. (2016), Kostic et al. (2013), Rubinstein et al. (2013)
ETBF	BFT (metalloprotease)	E-cadherin	E-cadherin cleavage → Wnt activation; NF-κB/STAT3 inflammation; SMOX → ROS	CMS4 mesenchymal subtype enrichment	Thomas et al. (2019), Goodwin et al. (2011), Chung et al. (2018), Dejea et al. (2018)
*P. anaerobius*	Surface adhesin	Integrin α2/β1	MDSC/TAM expansion → immunosuppression	Advanced adenomas, CRC	Long et al. (2019)
*C. freundii*	PreTA (DPD homolog)	5-FU	Direct fluoropyrimidine inactivation	Chemoresistance (pancreatic/colorectal)	Xu et al. (2025)

DSBs, double-strand breaks; ETBF, enterotoxigenic *Bacteroides fragilis*; BFT, *B. fragilis* toxin; ROS, reactive oxygen species; SMOX, spermine oxidase; CMS, consensus molecular subtype; MDSC, myeloid-derived suppressor cell; TAM, tumor-associated macrophage; 5-FU, 5-fluorouracil.

### 2.1 Genotoxicity and mutational signatures

One of the most profound paradigms shifts in contemporary oncology is the recognition that specific human commensal bacteria can function as classical, direct-acting carcinogens. A premier example is the pks^+^ strain of *E*. *coli*. This strain carries a 54-kilobase genomic island known as the polyketide synthase (pks) island, which encodes a highly complex enzymatic machinery composed of non-ribosomal peptide megasynthases and polyketide synthases (Nougayrède et al., 2006). The primary end-product of this enzymatic machinery is colibactin, a highly reactive, DNA-alkylating secondary metabolite. Due to its extreme chemical instability, colibactin cannot function as a secreted distant toxin. Instead, it requires direct physical contact between the live bacterium and the host apical epithelium for effective delivery.

Once translocated into the host cell nucleus, the electrophilic cyclopropane ring of colibactin directly alkylates adenine residues of the host DNA, forming bulky interstrand crosslinks (Bossuet-Greif et al., 2018). When the cellular replication machinery attempts to traverse these crosslinks, fork collapse ensues, producing severe DNA double-strand breaks (DSBs). This damage triggers robust phosphorylation of histone H2AX (γH2AX) and activation of the ATM/ATR DNA damage response pathways. Normal cells typically undergo apoptosis or permanent cell-cycle arrest (senescence) in response to such catastrophic damage. Cells harboring early precancerous mutations, such as loss of *APC*, often bypass these checkpoints, however, leading to rapid chromosomal instability (Arthur et al., 2012). Definitive validation of colibactin as a human carcinogen came using human colon organoids. Extended exposure of normal organoids to live pks^+^ *E. coli* yielded a distinct mutational signature defined by specific single base substitutions (SBS88) and small insertions and deletions (ID18) (Pleguezuelos-Manzano et al., 2020). Subsequent genomic profiling of large colorectal cancer cohorts identified this same colibactin-induced footprint in up to 20% of Western CRC cases, providing direct mechanistic evidence that colibactin accelerates the accumulation of somatic mutations required for early adenoma initiation (Pleguezuelos-Manzano et al., 2020).

Recent work has clarified the physiological drivers of pks^+^ *E. coli* colonization and colibactin production. The inflamed and neoplastic gut microenvironment, marked by altered oxygen gradients, increased iron availability, and mucus barrier defects, creates a permissive niche that favors pks^+^ *E. coli* over non-pathogenic commensals (Jans and Vereecke, 2025). This selective advantage persists despite the substantial metabolic cost of colibactin synthesis, fueling a self-perpetuating cycle in which bacterial genotoxicity exacerbates inflammation and inflammation, in turn, promotes further pathogen expansion. Beyond its canonical genotoxic effects, pks^+^ *E. coli* also contributes to cancer stemness, therapy resistance, and immune suppression, pointing to a broader oncogenic repertoire than initially appreciated (Jans and Vereecke, 2025).

### 2.2 Oncogenic signaling activation

While genotoxic bacteria initiate malignant transformation, other pathogens drive tumor proliferation and metastasis through direct manipulation of host signaling. *F. nucleatum*, an oral anaerobe linked to periodontitis, is a prominent example. Its enrichment in the colonic mucosa of CRC patients correlates consistently with advanced stage, increased metastasis, and poor prognosis (Brennan and Garrett, 2019). *F. nucleatum* exerts its oncogenic effects largely through specialized surface adhesins, the best characterized being *Fusobacterium* adhesin A (FadA). FadA forms an amyloid-like complex on the bacterial surface that binds the extracellular domain of E-cadherin, a transmembrane protein critical for epithelial tight junctions (Rubinstein et al., 2013). This binding compromises the intestinal barrier and, more importantly, triggers phosphorylation and internalization of the E-cadherin/β-catenin complex. Once internalized, β-catenin escapes its degradation complex and translocates to the nucleus, where it engages TCF/LEF transcription factors to hyperactivate Wnt signaling (Kostic et al., 2013). This upregulates oncogenes including *MYC*, *CCND1* (Cyclin D1), and matrix metalloproteinases, thereby driving epithelial hyperproliferation and tissue invasion.

In addition to FadA, *F. nucleatum* also deploys a second outer membrane protein, Fap2, which binds with high affinity to Gal-GalNAc, a carbohydrate moiety overexpressed on colorectal adenocarcinoma cells. This Fap2-Gal-GalNAc interaction accounts for the preferential tropism of *F. nucleatum* for the tumor microenvironment over adjacent healthy tissue (Abed et al., 2016). Recent structural studies have resolved the molecular basis of Fap2 binding to both cancer cells via Gal-GalNAc and immune cells via TIGIT, offering a framework for structure-guided inhibitor design (Schöpf et al., 2025). High-resolution genomic analyses have further prompted a shift to strain-level resolution in microbiome research. *F. nucleatum* is not functionally uniform. A distinct sub-lineage designated Clade 2 carries specialized genetic adaptations, enhanced acid tolerance and distinct metabolic capabilities, that permit it to survive gastric transit and selectively colonize the lower gastrointestinal tumor niche, identifying it as the primary oncogenic driver (Zepeda-Rivera et al., 2024; Ternes et al., 2020). This subspecies-level distinction has profound implications for diagnostic and therapeutic targeting, as not all *Fusobacterium* species possess equivalent pathogenic potential.

### 2.3 Toxin-mediated epithelial disruption

ETBF drives oncogenesis through secretion of the *B. fragilis* toxin (BFT), a 20-kDa zinc-dependent metalloprotease (Dejea et al., 2018). BFT cleaves the extracellular domain of E-cadherin on intestinal epithelial cells. This cleavage triggers intramembrane processing by host presenilin-1, a component of the γ-secretase complex, which releases the E-cadherin intracellular domain into the cytoplasm (Chung et al., 2018). As with *F. nucleatum*, loss of the extracellular domain liberates membrane-tethered β-catenin, leading to sustained Wnt pathway activation and the unchecked epithelial proliferation typical of early adenomas.

Beyond its direct effects on structural proteins, BFT is a potent activator of intracellular inflammatory cascades. Internalized BFT stimulates NF-κB and STAT3 signaling in epithelial cells, driving secretion of pro-inflammatory chemokines such as IL-8 that rapidly recruit neutrophils to the colonic mucosa. ETBF colonization also upregulates spermine oxidase, a polyamine catabolic enzyme whose overexpression generates intracellular hydrogen peroxide and reactive oxygen species that directly damage DNA and accelerate the accumulation of oncogenic mutations (Goodwin et al., 2011). This establishes a self-reinforcing cycle in which toxin-driven proliferation and elevated DNA damage provide a favorable environment for tumor evolution.

Recent studies have identified that ETBF plays a specific role in driving the mesenchymal subtype (CMS4) of CRC, the most aggressive molecular subtype characterized by stromal activation and poor prognosis. *B. fragilis* is significantly enriched in CMS4 tumors, and exposure of patient-derived organoids to ETBF induces CMS4-like features (Chang et al., 2025). This subtype-specific association may account for the poor outcomes seen in ETBF-high CRC patients and suggests that microbiome-directed therapies could be particularly effective in this molecular subgroup.

### 2.4 TIME remodeling

The survival and expansion of a nascent tumor heavily depend on its ability to evade host immune surveillance. Pathogenic gut microbiota plays an indispensable role in shielding colorectal tumors from immune detection by actively remodeling the tumor immune microenvironment (Bai et al., 2025).

*F. nucleatum* is particularly adept at immune subversion. The aforementioned Fap2 protein possesses a dual function. Aside from mediating tumor adherence, Fap2 directly binds to TIGIT (T cell immunoreceptor with Ig and ITIM domains), an inhibitory receptor highly expressed on the surface of human natural killer cells and cytotoxic CD8^+^ T cells (Gur et al., 2015). The binding of Fap2 to TIGIT effectively paralyzes these vital immune effectors, completely neutralizing their intrinsic tumor-killing capacity and allowing the tumor to expand unchecked. Concurrently, the inflammatory microenvironment generated by bacteria like ETBF and *P. anaerobius* actively recruits immunosuppressive cell populations. ETBF is renowned for orchestrating a dominant Th17 immune response. The massive local production of interleukin-17 not only promotes angiogenesis via VEGF upregulation but also strongly inhibits recruitment of anti-tumor CD8^+^ T cells (Wu et al., 2009). Similarly, *P. anaerobius* interacts with the host integrin α2/β1 receptor to heavily expand the population of myeloid-derived suppressor cells (MDSCs) and tumor-associated macrophages (TAMs). These recruited MDSCs secrete massive quantities of arginase and transforming growth factor-beta (TGF-β), creating an impenetrable immunosuppressive shield around the developing malignancy (Long et al., 2019).

### 2.5 Oncogenic modulation of microbial metabolites

Microbial metabolites add a further layer to colorectal oncogenesis. In the healthy colon, SCFAs such as butyrate function as tumor suppressors: they fuel normal colonocytes and trigger apoptosis in malignant cells. This protective role, however, becomes paradoxical in the presence of specific host mutations. The “butyrate paradox” describes how, in colonic epithelial cells carrying loss-of-function *APC* mutations, butyrate is diverted from its canonical tumor-suppressive pathways and instead oxidized as an energy source, thereby accelerating the proliferation of early adenoma cells (Belcheva et al., 2014). The outcome of butyrate exposure is therefore dictated by host genotype, underscoring the delicate interplay between microbial metabolites and the genetic landscape of the epithelium.

Secondary bile acids, particularly deoxycholic acid (DCA) and lithocholic acid (LCA), are more definitively pro-carcinogenic. These metabolites are generated exclusively by gut bacteria, notably *Clostridium* cluster XIVa and *Eubacterium* species, from primary bile acids secreted by the liver (Louis et al., 2014). A high-fat Western diet drives their overproduction. At elevated concentrations, DCA acts as a biological detergent, damaging cell membranes and inducing intracellular reactive oxygen species. It also activates the epidermal growth factor receptor (EGFR) and downstream MAP kinase cascades, suppressing p53-mediated apoptosis and promoting a hyperproliferative state (Yoshimoto et al., 2013). LCA contributes in parallel by engaging the vitamin D receptor and protein kinase C, reinforcing proliferative signaling and apoptotic resistance. This metabolic reprogramming of the intestinal niche thus links dietary factors, microbial metabolism, and host genomic instability. Recent evidence that secondary bile acids also modulate the tumor immune microenvironment—DCA, for instance, promotes the generation of immunosuppressive regulatory T cells—adds yet another dimension to their oncogenic repertoire and highlights the therapeutic opportunities in targeting microbial metabolite pathways.

## 3 Organoid platforms for host-microbe interaction research

The mechanistic discoveries detailed previously mandate an experimental platform capable of replicating the highly specialized physiological environment of the human gastrointestinal tract. While classical 2D cell cultures and murine models provided preliminary insights, they consistently failed to capture the architectural complexity and human-specific genomic fidelity required for definitive causal studies. The inception of 3D human intestinal organoids has revolutionized this landscape (Clevers, 2016). However, the initial iterations of these models presented significant topological and physiological barriers for microbiology. The subsequent bioengineering evolution of these platforms reflects a continuous effort to perfectly recapitulate the precise microenvironmental conditions necessary for studying microbial oncogenesis. Table 2 summarizes the evolution of organoid-based platforms for microbiome research.

**TABLE 2:** Evolution of organoid-based platforms for microbiome-CRC research.

Platform	Key features	Advantages	Limitations	Application
Classical 3D organoids (basal-out)	Lgr5^+^ ASC-derived; ECM-embedded; apical surface internal	Cellular fidelity; genetic stability; donor-specific	Microbial access barrier	Long-term epithelial biology
Microinjection	Direct bacterial injection into lumen	Preserves apical polarity; chronic exposure studies	Technically demanding; low throughput	Mechanistic host-pathogen studies
Apical-out organoids	Polarity inversion via ECM removal	High-throughput; seamless microbial inoculation	Reduced longevity; altered signaling	Bacterial adherence screening
2D organoid-derived monolayers	Transwell-supported; polarized access	TEER measurement; accessible apical/basal compartments	Loss of 3D architecture	Barrier function studies
Microfluidic Organ-on-Chip	Dual-channel; independent oxygenation; peristalsis	Resolves aerobic-anaerobic paradox; extended co-culture	Technical complexity; cost	Complex microbiome co-culture
ALI organoids	Air-exposed apical surface; preserved stroma	Native immune cell retention; stromal preservation	Limited to specific protocols	Tumor-immune-microbiome interaction
Multicellular co-culture	Patient-matched epithelial + immune + CAFs	Full TME reconstitution; personalized	Complexity; scalability challenges	Preclinical therapeutic testing

ASC, adult stem cell; ECM, extracellular matrix; TEER, transepithelial electrical resistance; ALI, air-liquid interface; TME, tumor microenvironment; CAF, cancer-associated fibroblast.

### 3.1 Structure and characteristics of human intestinal organoids

Human intestinal organoids are self-organizing microtissues derived from Lgr5^+^ adult stem cells embedded in a specialized extracellular matrix, most commonly Matrigel. When maintained in a defined culture cocktail containing Wnt, R-spondin, epidermal growth factor, and Noggin, these stem cells undergo multilineage differentiation and generate all major intestinal epithelial cell types, including absorptive enterocytes, mucin-producing goblet cells, antimicrobial Paneth cells, and enteroendocrine cells (Sato et al., 2011).

Despite their high cellular fidelity, conventional 3D intestinal organoids exhibit an inverted “basal-out” polarity. In this arrangement, the basal membrane contacts the surrounding extracellular matrix, whereas the apical surface lines a closed internal lumen. This structural feature creates substantial technical limitations for microbiome-related studies. *In vivo*, pathogenic bacteria such as *F. nucleatum* and pks^+^ *E. coli* specifically interact with the apical surface of intestinal epithelial cells. Direct addition of live bacteria to the external culture medium exposes the basal epithelial compartment to microorganisms—a non-physiological condition that typically elicits rapid and nonspecific epithelial apoptosis, rather than the sustained host–microbe interactions relevant to tumorigenesis.

### 3.2 Technical strategies for microbial interaction studies

To overcome these structural limitations, investigators have established high-precision microinjection approaches for intestinal organoids (Williamson et al., 2018). Using manual or robotic capillary systems, defined titers of live bacteria can be delivered directly into the sealed lumen, restricting microorganisms to the apical surface and supporting stable, long-term analyses of host–microbe interactions. This technical platform was critical for identifying colibactin-associated mutational signatures in primary human colonic epithelium, a process that cannot be faithfully modeled using conventional 2D cell lines (Pleguezuelos-Manzano et al., 2020). Although luminal microinjection remains the gold-standard method for modeling chronic microbial exposure, it requires extensive technical expertise, is labor-intensive, and is incompatible with high-throughput pharmacological or microbiological screening. To overcome these bottlenecks, several bioengineering strategies have been developed to expose the apical epithelial surface to the external culture environment.

A key innovation is the generation of “apical-out” polarity-reversed organoids. Brief removal from the extracellular matrix and suspension culture in specialized medium disrupts outside-in integrin signaling (Co et al., 2019), triggering spontaneous inversion of epithelial polarity. In this conformation, the apical brush border is exposed to the surrounding medium, allowing direct inoculation with complex bacterial communities. This setup supports scalable, high-throughput assays of bacterial adhesion, invasion, and toxin-dependent signaling without the need for microinjection.

Alternatively, researchers frequently utilize organoid-derived 2D monolayers. In this approach, mature 3D organoids are enzymatically dissociated into single cells and seeded onto porous Transwell inserts coated with a thin layer of human collagen or synthetic hydrogels (Marchini and Gelain, 2022). Unlike traditional immortalized cell lines, these primary monolayers preserve the genetic identity and cellular heterogeneity of the original patient donor. The transwell architecture establishes independent apical and basal compartments, making it an exceptionally powerful tool for measuring transepithelial electrical resistance (TEER). This approach is particularly valuable for quantifying the kinetics and magnitude of barrier dysfunction induced by bacterial virulence factors, such as the BFT toxin produced by ETBF.

### 3.3 Anaerobic–aerobic co-culture systems

A further and particularly formidable challenge in modeling the microbiome–CRC axis is the aerobic–anaerobic paradox. Human colonic epithelial cells depend on steady oxygen delivery from the vascularized basal lamina to support normal metabolism and viability. In contrast, most functionally important and oncogenic intestinal bacteria, including *F. nucleatum*, ETBF, and *P*. *anaerobius*, are strict obligate anaerobes. Under standard laboratory conditions, simultaneous culture of these two components is not feasible. Aerobic incubators rapidly induce oxidative death in obligate anaerobes, whereas fully anaerobic environments trigger lethal epithelial hypoxia within hours.

Organoid technology integrated with microfluidic Organ-on-a-Chip platforms has provided a powerful engineering solution to this longstanding dilemma (Jalili-Firoozinezhad et al., 2019). These microphysiological systems typically consist of two parallel microfluidic channels separated by a thin, porous, and flexible ECM-coated membrane. Intestinal epithelial cells differentiated from patient-derived organoids are cultured in the upper channel, while primary human microvascular endothelial cells are commonly seeded in the lower channel.

**FIGURE 1:**
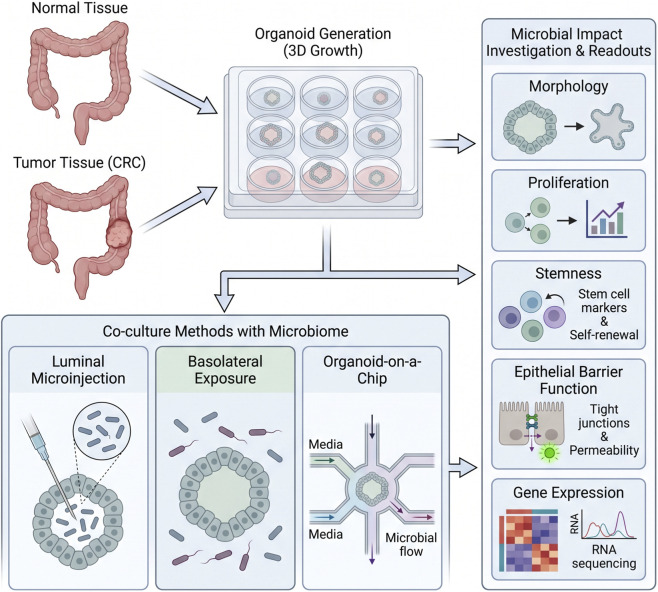
Overview of organoid models for CRC-microbiome research. This figure provides a comprehensive overview of the generation and application of colorectal organoid models in the context of microbiome research. It illustrates the derivation of organoids from normal or tumor tissues, their characteristic three-dimensional growth, and various methods for co-culturing with specific microbial species or complex communities. Key methodologies such as luminal microinjection, basolateral exposure, and integration into microfluidic “organoid-on-a-chip” systems are depicted. The figure highlights how these models enable the investigation of microbial impact on organoid morphology, proliferation, stemness, epithelial barrier function, and gene expression, providing a powerful platform for mechanistic dissection of host-microbe interactions in CRC.

A key advantage of this microfluidic design is the ability to independently regulate oxygen levels and environmental conditions in each channel. The basal endothelial channel can be continuously perfused with oxygenated medium to maintain epithelial viability, while the apical epithelial channel is flushed with anoxic medium or inert gas to establish an anaerobic lumen (Shah et al., 2016). This arrangement generates a sharp, physiologically relevant oxygen gradient that closely recapitulates the human colonic microenvironment. In addition, cyclic mechanical strain applied to the flexible membrane mimics intestinal peristalsis, which has been shown to boost mucin secretion and support more natural spatial organization of bacterial biofilms. Using these anaerobic Organ-on-a-Chip systems, investigators can now stably co-culture complex, viable fecal microbiomes from CRC patients with autologous organoids over extended periods (Figure 1) (Puschhof et al., 2021).

### 3.4 Reconstructed tumor microenvironment models

As previously elucidated, the oncogenic potential of the gut microbiota depends critically on its ability to modulate and evade the host mucosal immune system. Studying epithelial organoids in isolation therefore offers an incomplete view of TIME. The latest advance in organoid bioengineering centers on the systematic reconstruction of a comprehensive TME by integrating autologous immune cells and cancer-associated fibroblasts (CAFs).

To this end, investigators have established air-liquid interface (ALI) organoid culture systems that retain the native stromal architecture and endogenous immune cell populations present in primary tumor biopsies (Neal et al., 2018). In a widely adopted strategy, epithelial organoids and peripheral blood mononuclear cells (PBMCs) are separately isolated from the same colorectal cancer patient. By embedding patient-matched cytotoxic T lymphocytes and macrophages within the Matrigel surrounding epithelial organoids, researchers can directly visualize and quantify dynamic immune cell recruitment and functional suppression in real time (Dijkstra et al., 2018).

When key oncogenic pathogens such as *F*. *nucleatum* are introduced into these integrated models, studies can delineate how bacterial metabolites and surface adhesins, notably Fap2, directly impair the tumoricidal activity of autologous CD8^+^ T cells. This fully reconstituted, multi-cellular organoid platform not only validates core mechanisms of microbiota-driven immune evasion but also provides a physiologically relevant preclinical system for evaluating microbiome-targeted interventions and next-generation immunotherapies. Recent work has used this approach to confirm the roles of ETBF and *F. nucleatum* in activating Wnt/β-catenin and NF-κB signaling, thereby validating the functional significance of multi-omics-identified biomarkers (Wu et al., 2025).

### 3.5 Current technical limitations of organoid-based microbiome platforms

Despite the transformative progress outlined in preceding sections, organoid–microbiome co-culture systems still face substantial biological and engineering constraints that limit translational validity and experimental reproducibility. Critical assessment of these bottlenecks is essential to guide the next wave of bioengineering development.

Foremost among these challenges is the reproducibility of patient-derived organoids (PDOs). While PDOs accurately retain the genetic and phenotypic heterogeneity of primary tissues, this same fidelity introduces considerable experimental variation. Extended passaging of a single donor line promotes gradual transcriptional and phenotypic drift via clonal selection and *de novo* chromosomal alterations, weakening concordance with the original tumor (Lau et al., 2020). More critically, continued dependence on undefined, batch-variable biological reagents—most notably Matrigel and Wnt-conditioned medium—severely undermines inter-laboratory consistency; independent laboratories working with identical PDO lines often report discrepant transcriptomic profiles and drug-response phenotypes (Zhao Z. et al., 2022). Shifting toward fully defined, synthetic hydrogel matrices and recombinant morphogens thus represents an urgent priority for standardizing organoid-microbiome assays into reliable, high-fidelity platforms.

Beyond matrix-related variability, microfluidic Organ-on-a-Chip systems carry distinct economic and operational limitations. Although these devices effectively resolve the aerobic–anaerobic co-culture dilemma, broad implementation is hindered by high fabrication costs, demands for specialized engineering expertise, and inherently low throughput. Compounding these barriers is the near-universal use of polydimethylsiloxane (PDMS), which introduces a fundamental material confounder: the elastomer strongly absorbs small hydrophobic molecules, distorting pharmacokinetic readouts and measurements of metabolic crosstalk (Low et al., 2021). Operating even a modest set of parallel microfluidic models requires significant capital investment and labor-intensive handling, making current designs poorly suited for the high-throughput screening needs of modern drug discovery and large-scale microbiome research (Ingber, 2022).

Another major constraint is the limited long-term viability of primary human immune cells in co-culture. Even in optimized organoid-immune systems, tumor-infiltrating lymphocytes and myeloid cells typically show substantial functional decline within 7 days, severely narrowing the window for studying chronic host-microbe-immune crosstalk and testing immunomodulatory therapies.

Perhaps the most formidable hurdle remains the stable long-term maintenance of taxonomically and functionally complex, donor-matched whole fecal microbiota *in vitro*. Even within advanced anaerobic microfluidic chips, microbial communities undergo rapid compositional drift within 24–48 h: robust aerotolerant species outcompete fastidious obligate anaerobes, while key viral (virome) and fungal (mycobiome) components are largely lost under standard culture conditions, resulting in a truncated and functionally skewed microbiota (Yadav et al., 2023). In parallel, the progressive accumulation of bacterial fermentation by-products and host-derived cellular debris within the enclosed microenvironment rapidly induces epithelial cytotoxicity, yet the frequent medium exchanges required to alleviate this toxicity inadvertently destabilize the fragile microbial ecosystem and disrupt quorum-sensing dynamics (Jalili-Firoozinezhad et al., 2019). Achieving genuinely homeostatic, long-term microbiome-organoid co-culture therefore demands next-generation platforms that integrate real-time biochemical biosensors for metabolite monitoring, automated microfluidic waste clearance that preserves ecosystem integrity, and precisely calibrated multi-kingdom inoculation strategies capable of sustaining the full spectrum of bacterial, fungal, and viral constituents. Addressing these interconnected challenges will further require standardized dynamic microbiota inoculation protocols and multi-center validation studies to confirm the reproducibility of complex co-culture systems across diverse clinical cohorts.

## 4 Microbiome-derived biomarkers for CRC

**FIGURE 2:**
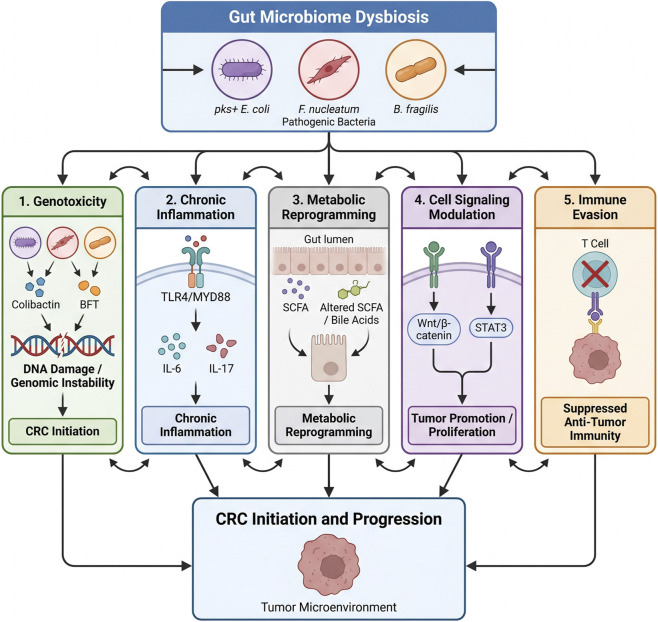
Key molecular mechanisms of microbiome-driven colorectal cancer. An intricate diagram illustrating the multifaceted molecular mechanisms through which gut microbiota contribute to colorectal cancer (CRC) initiation and progression. The figure details how specific pathogenic bacteria (e.g., *F. nucleatum*, pks^+^ *E. coli*, *B. fragilis*) exert their oncogenic effects through various pathways. These include the production of genotoxins (e.g., colibactin, BFT) leading to DNA damage and genomic instability; induction of chronic inflammation via TLR signaling (e.g., TLR4/MYD88) and subsequent cytokine cascades (IL-6, IL-17); metabolic reprogramming through altered short-chain fatty acid (SCFA) and secondary bile acid metabolism; direct modulation of host cell signaling pathways (e.g., Wnt/β-catenin, STAT3); and evasion or suppression of anti-tumor immune responses. The interplay between these mechanisms in shaping the tumor microenvironment and influencing CRC development is emphasized.

The rigorous mechanistic insights validated through advanced organoid platforms have catalyzed a rapid paradigm shift in the clinical management of colorectal cancer. The transition from fundamental microbiology to actionable clinical oncology primarily focuses on harnessing the microbiome as a highly sensitive, non-invasive reservoir for early diagnostics and as a robust independent predictor of patient prognosis and therapeutic response (Figure 2). Table 3 summarizes the leading microbial diagnostic and prognostic biomarkers.

**TABLE 3:** Microbial biomarkers for CRC diagnosis and prognosis.

Biomarker type	Specimen	Diagnostic/prognostic utility	Sensitivity/specificity	Validation status	References
*F. nucleatum* DNA (qPCR)	Stool	Early adenoma detection	AUC 0.78–0.85	Multi-cohort	Wang and Fang (2023), Zarour and Trinchieri (2025), Yu et al. (2024)
*F. nucleatum* + FIT combination	Stool	Advanced adenoma sensitivity improvement	+25%–30% vs. FIT alone	Clinical validation	Wang and Fang (2023)
*pks* island detection	Stool/Feces	EOCRC risk stratification	Variable	Cohort-specific	Akimoto et al. (2021), Pleguezuelos-Manzano et al. (2020)
Multi-kingdom signature (14 microbial +8 protein markers)	Tissue	Adenoma vs. carcinoma discrimination	AUROC 0.962	External validation (2 cohorts)	Wu et al. (2025)
Intratumoral *F. nucleatum* load	Tissue	Overall survival; metastasis prediction	HR ∼2.5–4.0 for poor outcome	Multiple retrospective studies	Ren et al. (2025), Bullman et al. (2017), Spaander et al. (2023)
CMS4-associated *B. fragilis* enrichment	Tissue	CMS4 subtype identification; poor prognosis	LDA score 4.7	Independent validation	Chang et al. (2025)

AUC, area under curve; FIT, fecal immunochemical test; EOCRC, early-onset colorectal cancer; CMS, consensus molecular subtype; LDA, linear discriminant analysis; HR, hazard ratio.

### 4.1 Non-invasive early screening for CRC

Effective clinical management of CRC is fundamentally dependent on early detection. Although optical colonoscopy remains the gold-standard approach for diagnosis and resection of precancerous adenomas, broader application as a population-level screening tool is limited by its invasive nature, substantial healthcare costs, and suboptimal patient adherence. Current non-invasive options, led by the fecal immunochemical test (FIT), rely on detection of occult blood loss. However, FIT shows limited sensitivity for early asymptomatic adenomas, as these early lesions rarely produce bleeding (Wong and Yu, 2019). There is therefore an urgent clinical need for sensitive, stage-specific biomarkers. The gut microbiome undergoes marked, functionally consistent shifts long before tumors become clinically apparent, offering a uniquely promising foundation for next-generation non-invasive diagnostics.

Fecal microbiome DNA-based testing has emerged as a leading strategy in precision oncology. High-throughput shotgun metagenomic sequencing across ethnically and geographically diverse cohorts has defined a globally reproducible microbial signature linked to colorectal neoplasia. This signature consistently features enrichment of key pathogenic taxa, including *F. nucleatum*, *P. anaerobius*, *Parvimonas micra*, and *Porphyromonas asaccharolytica* (Wirbel et al., 2019). Targeted qPCR assays quantifying fecal *F. nucleatum* levels have demonstrated strong diagnostic performance, and clinical validation studies confirm that combining *F. nucleatum* measurement with conventional FIT significantly improves detection of advanced precancerous adenomas relative to FIT alone (Wang and Fang, 2023; Zarour and Trinchieri, 2025; Yu et al., 2024). This combinatorial approach addresses a critical gap in early CRC detection. In addition, targeted screening for bacterial virulence loci, such as the pks island in genotoxic *Escherichia coli* or enterotoxin genes in *Bacteroides fragilis*, provides a predictive strategy for identifying younger individuals at elevated risk of early-onset CRC (Jans and Vereecke, 2025).

To improve diagnostic precision and reduce biological variability, translational research has increasingly shifted from single-marker DNA detection toward integrated multi-omic strategies. Combining metabolomic profiling with metagenomics substantially improves the AUC of diagnostic prediction models. By quantifying microbially modified metabolites in feces or serum, the functional output of dysbiotic communities can be translated into clinically usable classifiers. Integrated analyses show that elevated ratios of secondary bile acids, altered polyamine levels, and distinct branched-chain amino acid profiles strongly correlate with advanced adenomas (Chen et al., 2022). Recent advances have further combined tissue-based metagenomics and proteomics, identifying 14 microbial multi-kingdom markers and eight protein markers that clearly distinguish adenomas from carcinoma, with an AUROC of 0.962 and successful external validation (Wu et al., 2025).

Development of robust, clinically deployable predictive models from high-dimensional compositional data requires rigorous computational frameworks. Multiple machine learning approaches have been validated for CRC-associated microbial classification. Ensemble methods, particularly Random Forest and XGBoost, deliver strong performance due to their ability to model non-linear relationships and complex feature interactions while generating interpretable importance scores (Knights et al., 2011; Pasolli et al., 2016). Support Vector Machines with radial basis function kernels represent a useful alternative, especially in high-dimensional settings where feature numbers approach or exceed sample sizes—a common challenge in metagenomic studies (Topçuoğlu et al., 2020).

Notably, microbial relative abundance data are inherently compositional, with values constrained to a fixed total (e.g., 100%), creating statistical dependence and increased risk of spurious correlations. Centered log-ratio (CLR) transformation resolves this constraint by expressing each taxon relative to the sample geometric mean, projecting data into an unconstrained Euclidean space compatible with standard statistical and machine learning workflows (Gloor et al., 2017; Quinn et al., 2018). Failure to apply such transformations can lead to inflated false discovery rates and poor reproducibility. When CLR-transformed microbial features are combined with normalized metabolomic and proteomic data within ensemble pipelines, models regularly achieve sensitivity and specificity above 90% for distinguishing early-stage CRC from healthy controls (Quinn et al., 2018; Cammarota et al., 2020). Machine learning frameworks built on integrated multi-omic datasets thus represent a key translational advance, converting complex biological signatures into standardized, interpretable tools to support clinical decision-making.

### 4.2 Prognostic stratification for CRC

Beyond serving as a primary diagnostic tool, the microbiome harbors profound prognostic value for patients who have already been diagnosed with malignancies. The concept of the “intratumoral microbiome” has gained immense clinical traction. Rather than merely residing in the colonic lumen, specific bacteria actively invade and establish stable communities deeply within the malignant tissue matrix.

Extensive retrospective histological and genomic analyses have established a powerful inverse correlation between the intratumoral load of *F. nucleatum* in surgically resected tissues and overall patient survival. Colorectal tumors exhibiting high *F. nucleatum* colonization rates are clinically associated with an aggressively invasive phenotype, a significantly higher propensity for local lymph node metastasis, and accelerated disease recurrence (Ren et al., 2025; Bullman et al., 2017; Spaander et al., 2023). Strikingly, studies utilizing fluorescent *in situ* hybridization (FISH) have demonstrated that live *F. nucleatum* cells frequently travel alongside migrating circulating tumor cells (CTCs) to distant metastatic sites, most notably the liver (Jiang et al., 2026). This microbial persistence in hepatic metastases suggests that measuring the bacterial load of the primary tumor can reliably stratify patients into high-risk categories, thereby guiding oncologists in the precise escalation of adjuvant therapeutic strategies following surgical resection.

### 4.3 Prediction of chemoresistance

Perhaps the most critical clinical implication of the tumor microenvironment is the active role gut microbiota play in dictating the efficacy of conventional chemotherapeutic regimens. The pervasive phenomenon of chemoresistance remains the primary cause of treatment failure and mortality in advanced colorectal cancer. Groundbreaking translational studies have mechanistically proven that intratumoral bacteria actively shield cancer cells from pharmacological intervention.

*F. nucleatum* serves as the archetypal mediator of this resistance mechanism. High intratumoral abundance of *F. nucleatum* is causally linked to profound clinical resistance to both 5-fluorouracil (5-FU) and oxaliplatin, the foundational pillars of colorectal chemotherapy. At the molecular level, *F. nucleatum* adheres to the cancer cell surface and strongly activates the Toll-like receptor 4 (TLR4) and the downstream MYD88 signaling cascade (Dadgar-Zankbar et al., 2024; Yu et al., 2017; Zhao T. et al., 2022). This specific receptor activation subsequently triggers a highly coordinated intracellular autophagic response. By upregulating autophagy, the malignant cells can efficiently degrade and clear the damaged cellular components and reactive oxygen species generated by the chemotherapeutic agents, thereby evading drug-induced apoptosis (Haider et al., 2020; Khan et al., 2024).

Furthermore, beyond intracellular signaling manipulation, diverse commensal species possess the intrinsic enzymatic machinery required to directly metabolize and inactivate circulating systemic drugs before they can reach the tumor bed (Alexander et al., 2017). Recent research has identified that *Citrobacter freundii* harbors the preTA operon encoding a functional homolog of human dihydropyrimidine dehydrogenase, enabling direct inactivation of 5-FU (Xu et al., 2025). This mechanism, initially characterized in pancreatic cancer, has broader implications for colorectal cancer where similar intratumoral bacteria may drive fluoropyrimidine resistance. Importantly, the clinically approved dihydropyrimidine dehydrogenase inhibitor gimeracil effectively blocks bacterial PreTA activity, preserving 5-FU efficacy and offering a potential strategy to overcome this form of chemoresistance (Xu et al., 2025). Comprehensive profiling of a patient’s baseline microbiome is rapidly becoming an indispensable prerequisite for the future of personalized oncology, holding the ultimate promise of tailoring chemotherapy regimens specifically to circumvent an individual’s unique microbial resistance profile.

### 4.4 Clinical translation challenges

Despite the compelling diagnostic potential of multi-kingdom microbial and protein marker panels demonstrated in discovery cohorts (Wu et al., 2025), substantial translational barriers currently limit their application as regulated clinical laboratory tests. To achieve the reproducibility and robustness required for routine diagnostic use, rigorous standardization is needed across the entire analytical pipeline, from pre-analytical sample handling to bioinformatic classification.

Pre-analytical factors are the main source of inter-laboratory variability. The composition of fecal and tissue microbiomes is highly sensitive to collection methods, preservative types, storage temperatures, and freeze-thaw cycles, yet universally accepted standard operating procedures for clinical microbiome testing are still lacking (Sinha et al., 2017). In tissue-based diagnostics, prolonged warm ischemia and formalin fixation cause differential degradation of DNA from Gram-negative bacteria, potentially distorting relative abundance profiles. In addition, the DNA extraction step introduces two major confounding factors: first, different commercial kits exhibit inherent extraction biases due to varying lysis efficiency for Gram-positive bacteria, fungi, and spores (Tourlousse et al., 2021); second, trace microbial DNA present in extraction reagents and consumables, known as the “kitome”, can dominate the signal in low-biomass tissue samples, leading to false-positive taxonomic assignments that seriously compromise diagnostic specificity (Salter et al., 2014).

After nucleic acid extraction, sequencing depth and platform choice impose additional analytical constraints. Low-depth shotgun metagenomic sequencing, while cost-effective, often fails to detect low-abundance pathogens and clinically important virulence genes such as the pks island. In contrast, ultra-deep sequencing faces prohibitive throughput and cost limitations for high-volume clinical laboratories (Amos et al., 2020). Moreover, different sequencing platforms and library preparation kits introduce batch effects that interfere with cross-cohort comparisons and mask true biological signals. Finally, reliable bioinformatic analysis requires consensus reference databases, validated taxonomic classifiers, and strict quality control thresholds to filter contaminants and correct for technical covariates. The lack of a universally accepted bioinformatic framework, combined with a critical shortage of certified clinical metagenomic reference materials, often leads to inconsistent microbial signatures when the same clinical sample is analyzed using different pipelines (Huttenhower et al., 2023; Isali et al., 2024).

Addressing these standardization challenges is an urgent clinical need. Although multi-center initiatives such as the Microbiome Quality Control project have established basic guidelines, oncology-specific adaptations, including tumor-specific DNA extraction controls, spike-in standards for absolute quantification, and clinical accreditation of metagenomic workflows, remain at an early stage. Until these methodological obstacles are systematically resolved, the translation of multi-kingdom marker panels from proof-of-concept studies to regulated and reimbursable clinical diagnostics will remain an aspirational goal rather than a clinical reality.

## 5 Precision therapeutic strategies targeting oncogenic microbiota

The realization that specific intestinal microbiota actively drive oncogenesis and mediate therapy resistance has necessitated a radical evolution in precision oncology. Rather than merely treating the host cells, modern clinical strategies must now actively neutralize microbial oncogenic drivers and deeply remodel the local ecosystem. This therapeutic imperative has catalyzed the development of highly sophisticated, microbiome-targeted interventions encompassing precision antimicrobials, viral predators, ecological reconstitution, and advanced synthetic biology. Table 4 summarizes key preclinical and clinical advances.

**TABLE 4:** Emerging microbiome-targeted therapeutic strategies for CRC.

Strategy	Agent/platform	Target	Mechanism	Development stage	Key finding	References
Precision antimicrobial	FadA-binding small molecules	*F. nucleatum* FadA	Blocks E-cadherin binding without bacterial killing	Preclinical	Inhibits Wnt signaling; preserves microbiota	Rubinstein et al. (2013), Schöpf et al. (2025), Pan et al. (2023)
Bacteriophage therapy	*F. nucleatum*-specific phages	*F. nucleatum*	Lytic elimination of specific strains	Preclinical	Reduces tumor burden in mouse models	Zheng et al. (2019), Joo et al. (2024), Kowsarkhizi et al. (2025)
Phage-guided nanotechnology	Chemophore-conjugated phages	*F. nucleatum* in TME	Targeted cytotoxic delivery; bacterial lysis	Preclinical	Maximizes intratumoral drug concentration	Zheng et al. (2019), Shuwen and Kefeng (2022)
FMT for ICB sensitization	Healthy donor or responder FMT	Gut microbial ecosystem	Restores immunostimulatory microbiota (e.g., *Bifidobacterium*, *Akkermansia*)	Phase I/II trials	Converts non-responders to responders in melanoma; CRC trials ongoing	Tjalsma et al. (2012), Baruch et al. (2021), Lin et al. (2023), Wang et al. (2024), Hajjar et al. (2026), Jamal et al. (2023)
Engineered probiotics	Cytokine-secreting *E. coli* Nissle	Hypoxic tumor niche	Local production of immunomodulators	Preclinical	Induces durable tumor regression	Chowdhury et al. (2019)
Checkpoint nanobody delivery	*E. coli* Nissle with anti-PD-L1 nanobody	Tumor-localized ICB	Continuous local checkpoint inhibition	Preclinical	Reduces systemic toxicity	Gurbatri et al. (2020)
Nisin G-producing probiotic	*S. salivarius* DPC6487	*F. nucleatum*	Bacteriocin-mediated narrow-spectrum inhibition	Preclinical	Controls *F. nucleatum* in *ex vivo* colon model	Lawrence et al. (2025)

FMT, fecal microbiota transplantation; ICB, immune checkpoint blockade; TME, tumor microenvironment.

### 5.1 Narrow-spectrum virulence inhibitors

Historically, the administration of broad-spectrum systemic antibiotics was considered a potential strategy to clear intratumoral bacteria. However, this indiscriminate approach has proven clinically detrimental. The non selective eradication of both pathogenic drivers and vital beneficial commensals completely collapses mucosal homeostasis, frequently exacerbating intestinal inflammation and accelerating tumor progression (Wang and Ding, 2025). Furthermore, extensive antibiotic exposure severely compromises the efficacy of immune checkpoint inhibitors by depleting the systemic microbial signals required for basal immune priming (Simpson et al., 2023; Joachim et al., 2023).

Consequently, contemporary pharmacological efforts have pivoted toward highly specific, narrow spectrum eradication strategies. Instead of utilizing conventional bactericidal agents, researchers are developing custom small molecule inhibitors designed to selectively neutralize exact bacterial virulence factors without affecting microbial viability. For example, novel experimental compounds specifically bind and block the amyloid like structures of the FadA adhesin, physically preventing *F. nucleatum* from docking onto host E-cadherin receptors (Rubinstein et al., 2013; Schöpf et al., 2025; Pan et al., 2023). By precisely disarming the pathogen rather than blindly annihilating the microbiome, clinicians can halt oncogenic Wnt signaling while perfectly preserving the global ecological balance of the gut.

A particularly promising natural product approach has recently emerged from screening gut-derived bacterial strains for anti-*F. nucleatum* activity. *Streptococcus salivarius* DPC6487 produces a novel nisin variant designated nisin G, which exhibits narrow-spectrum activity against *F. nucleatum* while exerting minimal impact on the surrounding microbiota in an *ex vivo* model of the human distal colon (Lawrence et al., 2025). This bacteriocin-based strategy offers a targeted approach that leverages the natural antimicrobial repertoire of commensal bacteria.

### 5.2 Strain-specific bacteriophage therapy

To achieve absolute eradication specificity, the field of translational microbiology has revitalized the application of lytic bacteriophages. Phages are naturally occurring viruses that infect and lyse bacterial hosts with extreme taxonomic precision, often limited to a single specific bacterial strain. By meticulously isolating and propagating phages that exclusively target keystone oncogenic drivers like *F. nucleatum* or pks^+^ *E. coli*, researchers can engineer hyper specific microbial interventions that leave the healthy surrounding commensal populations entirely undisturbed (Joo et al., 2024; Kowsarkhizi et al., 2025).

Beyond acting as simple biological eradicators, bacteriophages are currently being bioengineered as highly advanced, guided nanotherapeutics. Recent breakthroughs in nanotechnology have enabled scientists to conjugate powerful chemotherapeutic molecules or metallic nanoparticles directly to the viral capsid (Zheng et al., 2019; Shuwen and Kefeng, 2022). When administered orally or intravenously, these modified phages actively seek out their target bacteria dwelling deep within the hypoxic core of the tumor. Upon successful binding and lysis, the phages simultaneously release their lethal cytotoxic payload directly into the TME. This extraordinary phage guided drug delivery mechanism dramatically maximizes local drug concentration while completely mitigating systemic chemotherapeutic toxicity.

### 5.3 Fecal microbiota transplantation

While phages address specific pathogenic overgrowth, holistic ecological reconstitution requires the introduction of entire functional microbial communities. FMT has profoundly revolutionized the clinical management of refractory intestinal infections and is currently undergoing rigorous evaluation as a transformative adjunct in oncology (Hajjar et al., 2026; Jamal et al., 2023). Its primary oncological application lies in overcoming immunotherapy resistance, though the evidence base varies by cancer type.

Immune checkpoint inhibitors, particularly those targeting the PD-1 and PD-L1 pathways, exhibit notoriously variable efficacy among CRC patients (Lin et al., 2023; Wang et al., 2024). Groundbreaking clinical trials have definitively demonstrated that the composition of the baseline gut microbiome dictates this immunological response. FMT has demonstrated preliminary clinical efficacy in boosting anti-PD-1 responses in patients with metastatic melanoma. In a landmark proof-of-concept trial (NCT03353402), FMT from durable immunotherapy responders converted a subset of refractory melanoma patients into clinical responders, with responder-derived engraftment of immunostimulatory consortia systemically revitalizing dendritic cell antigen presentation and rescuing cytotoxic T cell exhaustion within the TIME (Baruch et al., 2021). The successful engraftment of these specific beneficial consortia (often highly enriched in *Bifidobacterium* and *Akkermansia* species) systemically revitalizes dendritic cell antigen presentation and rescues cytotoxic T cell exhaustion within the TIME (Zeng et al., 2019; Mafe and Büsselberg, 2025). However, it is imperative to emphasize that robust clinical data for FMT in colorectal cancer remain absent. These melanoma-derived findings have not yet been replicated in gastrointestinal malignancies, and a phase II trial evaluating FMT plus pembrolizumab in MSI-H/dMMR colorectal cancer is currently ongoing (NCT04691031), with its results eagerly awaited to determine cross-cancer applicability (Du et al., 2025). The selection of rigorously screened healthy donors, rather than donor selection based on extreme responder status, offers a more feasible and potentially safer alternative for large-scale clinical application, yet this strategy remains untested in CRC (Du et al., 2025).

However, the application of FMT in oncology populations demands heightened safety vigilance. CRC patients undergoing chemotherapy or immunotherapy frequently exhibit compromised intestinal barrier integrity and systemic immunosuppression, rendering them vulnerable to translocation of donor-derived pathobionts and consequent opportunistic infections or, in rare cases, sepsis (Defilipp et al., 2019). Multiple consensus guidelines now mandate rigorous donor screening protocols, including extended microbiological and virological panels, and recommend against FMT during periods of severe neutropenia or active mucosal injury. These safety constraints underscore a critical therapeutic gap: while FMT effectively reconstitutes a health-associated microbial ecosystem, its inherent biological complexity and potential for unpredictable engraftment in immunocompromised hosts limit its routine deployment in oncology. Furthermore, the complete absence of CRC-specific efficacy data, coupled with the pending results of ongoing trials, means that FMT for CRC immunotherapy sensitization remains an investigational strategy rather than an established clinical modality. This limitation has directly motivated the development of more precisely controllable alternatives, including bacteriophage-guided precision antimicrobials and genetically engineered probiotics with defined mechanisms of action, which are discussed in the following sections.

### 5.4 Next generation engineered probiotics

The ultimate frontier of microbiome targeted therapeutics transcends naturally occurring bacteria through the revolutionary lens of synthetic biology. Rather than relying on the intrinsic properties of natural strains, scientists are extensively genetically reprogramming commensal bacteria to function as autonomous, living bio therapeutics. These engineered probiotics are specifically designed to exploit the unique physiological parameters of solid tumors, such as local hypoxia and necrotic accumulation, ensuring they exclusively colonize the tumor bed rather than healthy organs (Muhammad et al., 2026).

Once localized within the malignancy, these living medicines execute complex genetic circuits. Certain programmable strains are engineered to continuously synthesize and secrete potent immunomodulatory cytokines directly into the local microenvironment (Chowdhury et al., 2019). Of particular note, an *E*. *coli* Nissle 1917 strain engineered to secrete anti-PD-L1 nanobodies significantly inhibited tumor growth (Gurbatri et al., 2020). However, the human safety, pharmacokinetics, and clinical activity of probiotic-based local checkpoint delivery remain entirely unevaluated (Gurbatri et al., 2020). Other synthetic variants release decoy receptors that neutralize oncogenic toxins like BFT, acting as living mucosal biosensors. While these proof-of-concept studies highlight extraordinary therapeutic potential, all engineered probiotic strategies for CRC are currently at the preclinical stage and require rigorous toxicological and clinical validation before human translation.

## 6 Conclusion and future perspectives

The conceptual transition of the gut microbiome from a passive bystander to a central, actionable protagonist in colorectal carcinogenesis represents one of the most significant medical breakthroughs of the 21st century. This review has highlighted the critical shift from broad taxonomic associations to highly specific molecular paradigms. Notably, the identification of *F. nucleatum* Clade 2 as the dominant CRC-associated lineage, the capacity of *E. coli*-derived CNF1 to induce fetal reprogramming in intestinal stem cells, and the role of ETBF in driving the aggressive CMS4 subtype collectively demonstrate that microbial pathogenesis extends far beyond simple inflammation or DNA damage to include the direct subversion of host developmental pathways.

A major catalyst for these mechanistic breakthroughs has been the rapid evolution of preclinical models. By overcoming the limitations of traditional aerobic-anaerobic co-culture, bioengineered organoids and microfluidic Organ-on-a-Chip platforms now provide the high-fidelity environments required to dissect host-microbe interactions. These systems successfully bridge the gap between *in vitro* simplifications and *in vivo* complexities, establishing a rigorous structural foundation for validating bacterial virulence and testing targeted interventions.

Translating this deep biological understanding into clinical practice constitutes the next primary objective. Diagnostic frameworks are increasingly benefiting from multi-omics integration, where high-resolution microbial signatures demonstrate exceptional accuracy in early, non-invasive CRC detection. Concurrently, the finding that specific intratumoral bacteria actively metabolize chemotherapeutic agents, such as the enzymatic degradation mediated by *E. coli*, identifies the microbiome as a major variable in treatment efficacy. Addressing this microbiome-mediated resistance through selective microbial ablation or adjuvant enzyme inhibitors is rapidly emerging as a viable clinical strategy.

Looking forward, the synergy between global multi-omics datasets and artificial intelligence will be essential for distilling complex microbial data into universally applicable clinical biomarkers. As precision oncology continues to mature, we anticipate that patient-specific microbiome profiling will transition from an exploratory research tool to a standard clinical prerequisite (Wei et al., 2023; Zhang et al., 2025; Mukherjee et al., 2025; Sepich-Poore et al., 2021). Ultimately, through the integration of synthetic biology and rationally engineered living biotherapeutics, the field is poised to progress from merely observing microbial dynamics to actively programming the gut ecosystem as a targeted therapeutic modality against CRC.
